# Shame Withdraws, Guilt Corrects: Distinguishing Shame and Guilt in Goal Pursuit—An Experimental Study

**DOI:** 10.3390/bs15060725

**Published:** 2025-05-24

**Authors:** Rania W. Semaan

**Affiliations:** School of Business Administration, American University of Sharjah, Sharjah P.O. Box 26666, United Arab Emirates; rsemaan@aus.edu

**Keywords:** goal pursuit, goal failure, shame, guilt, goal-directed behavior

## Abstract

Research has shown that much of consumer behavior is goal-directed. However, such behavior is accompanied by emotions that guide goal-directed effort. This research aims to explain how two equally valenced emotions, shame and guilt, arising from goal violation, could lead to different allocations of effort toward goal attainment. Building on emotion attribution theory, I report one experiment that demonstrates the underlying mechanisms of shame and guilt and how they differ in their effect on subsequent goal-directed behavior. Results indicated that individuals with high shame proneness and low guilt proneness and who are primed with shame chose to violate their goal, providing evidence that shame is maladaptive and destructive in nature, leading consumers to withdraw from their goals.

## 1. Introduction

Much of consumer behavior is goal-directed (Bagozzi & Dholakia, 1999). For example, consumers’ spending decisions are affected by their health, ethical, sustainability, and investment goals. Hence, goals are important and play a central role in understanding consumer behavior. A goal is a desired outcome that enters the mind of a decision-maker as a mental representation associated with affect, toward which action may be directed (Pervin, 1989). This research focuses on the emotions that accompany goal-directed behavior and how these emotions influence people’s subsequent efforts toward a certain goal attainment. Although emotional reactions to goal progress have previously been associated with subsequent goal-related behavior, much of this literature has examined these relations at the level of affect (valenced states of long duration and low intensity that occur gradually without a specific immediate cause, Schwarz & Clore, 1996). However, Herrald and Tomaka (2002) show that discrete emotions lead to different behaviors, even if they share the same valence. Therefore, two equally valenced but different discrete emotional reactions to goal progress could lead to two different subsequent goal-related behaviors. In this research, the focus is on two emotions that arise while evaluating oneself: Shame and Guilt. Previous research (Tangney, 1990; Tangney et al., 1992; Niedenthal et al., 1994) distinguished between these two negative emotions. Even though shame and guilt may arise from the same situation, they can lead to two different actions. Shame involves negative feelings about the stable, global self, where the individual who is experiencing shame is concerned with an evaluation of the self (Leach & Cidam, 2015), whereas guilt involves negative feelings about a specific behavior, action, or transgression taken by the self (Mróz & Sornat, 2022). For example, consider a person who has vowed to purchase sustainable products, but in one instance, she failed to do so. If she blames her failure on her irresponsibility and inability to make ethical choices, then she is attributing her failure to the self (irresponsible) and, therefore, feels ashamed. Whereas, if she blames her failure on the fact that she was in a hurry and did not take the time to check the product’s sustainability credentials, then she is placing the blame on an action taken by herself (not researching) and, therefore, feels guilty. However, in the behavior and indulgence literature, guilt has been shown to play an important role in choices and self-control (Khan et al., 2005). For example, guilt has been studied as an emotional experience where priming people with guilt leads to a guilty experience and, hence, greater self-control and less indulgence (Zemack-Rugar et al., 2007; Giner-Sorolla, 2001). On the other hand, guilt has also been studied as a motivational state where guilt primes motivate consumers to interpret their decisions as virtuous in order to avoid guilt, and feeling virtuous can paradoxically lead to more indulgence (Khan et al., 2009). However, the same distressing situation could cause different responses, namely, shame or guilt (Tangney, 1992), and, therefore, generate different behaviors (Herrald & Tomaka, 2002). While there is ample research on the consequences of guilt on consumption (Saintives & Lunardo, 2016), the findings are mixed, linking guilt to both reparative action and avoidance. In contrast, research on the consequences of shame on consumption is surprisingly scarce, particularly in the context of goal-directed behavior. The few studies that have included shame report inconsistent and sometimes contradictory results (Leach & Cidam, 2015). While some suggest that shame and guilt are functionally similar, often combining them or not clearly distinguishing between the two emotions (e.g., Saintives & Lunardo, 2016), others have highlighted their differences (e.g., Tangney et al., 2007). Even among studies that have distinguished between the two emotions, some portrayed shame as constructive (Lickel et al., 2014), while others have characterized it as avoidant (Tangney et al., 1992). This has left a significant gap in our understanding of how shame operates distinctly from guilt, particularly in the realm of consumer behavior. The current study seeks to extend existing research by explicitly distinguishing between shame and guilt and examining their distinct effects in a goal-directed consumption context, thus clarifying their unique behavioral consequences and roles in self-regulation following goal failure. We are now in a period where shaming is employed more frequently by several entities, ranging from individuals and business owners to government agencies. Therefore, it is crucial to understand how experiences of shame, distinct from guilt, influence individuals’ goal pursuit. This research directly addresses this gap by focusing on the unique role of shame in shaping goal-directed behavior.

Therefore, I propose that the discrete type of emotion, rather than the valence of the emotion, which flows from an ongoing goal pursuit, influences the effort allocated to goal attainment. Specifically, the objective of this paper is to examine how shame versus guilt as a result of goal failure can affect whether people strive harder toward their goal or simply choose to withdraw and violate that goal. More importantly, the primary focus of this research is to explore whether goal failure can elicit feelings of shame and to examine the role of shame in influencing subsequent behavior. It is hypothesized that if goal failure induces shame rather than guilt, individuals will internalize the failure as a reflection of personal inadequacy (e.g., feeling incompetent). This internalized shame is expected to lead to an avoidance motivation, where individuals seek to distance themselves from the source of their shame. Consequently, this reaction may impair their future performance and hinder progress toward goal attainment.

This article proceeds as follows. First, I briefly review the literature on goal progress and its effect on subsequent behavior. Next, I discuss the distinction between shame and guilt. Sections three and four describe the experiment and results, followed by a discussion and implications.

## 2. Theoretical Background

### 2.1. Goal Progress

A broad literature suggests that goals motivate individuals and make them strive harder to accomplish a task. However, the goal progress literature offers opposing predictions about goal-directed behavior. For example, individuals referring to their activities as failures were more demotivated and showed less goal commitment and, consequently, lower performance (Bandura & Simon, 1977). In a similar vein, researchers argue that when people violate a certain goal, they are more likely to engage in the previously constrained activity that prevents goal attainment and leads to deterioration in subsequent performance (Soman & Cheema, 2004). Previous literature on goal-directed behavior shows that if individuals fail to accomplish their goal, they are expected to engage in ruminative thought (Jones et al., 2013) and experience negative emotions (Heath et al., 1999). Hence, negative emotions that signal an unfavorable event reinforce avoidance behaviors because they activate the avoidance system and, therefore, lower goal performance (Ilies & Judge, 2005). This failure can be damaging to consumers’ self-esteem and have an adverse effect on their motivation while setting future-related goals (Höpfner & Keith, 2021; Y. Shi et al., 2013; Welsh et al., 2020).

In contrast, Fishbach and Dhar (2005) demonstrate that individuals who perceive themselves as having made limited progress toward their goal are more likely to engage in goal-consistent behavior, while those who perceive substantial progress are more inclined to pursue goal-inconsistent behaviors. This finding suggests that negative emotions stemming from goal failure signal slower-than-expected progress, motivating individuals to exert greater effort and adjust their behavior to align with their goals (Carver & Scheier, 1990, 1998; Lawrence et al., 2002). Other researchers found no evidence of goal failure in future-related behaviors despite experiencing negative emotions and low self-esteem (Lebeau et al., 2018). As a result, previous research offers contrasting views on how negative emotions following goal failure influence subsequent behaviors. This article seeks to shed light on this issue. Unlike general affective states, discrete emotions are intense responses to specific events (Schwarz & Clore, 1996). This makes them particularly relevant for exploring the relationship between the emotions that arise from goal failure and subsequent behavior. Several arguments support this rationale. First, each discrete emotion reflects a distinct interaction between an individual and their environment with its own unique pattern of cognitive appraisals (Frijda, 1986, 1987; Lazarus, 1991; Scherer, 1984). Second, people tend to use different coping strategies in response to different discrete emotions. For example, people cope differently with guilt than they do with shame (Tangney, 1995). Where guilt is associated with active attempts to rectify a bad situation, shame is associated with avoidance and the motivation to disappear. Therefore, shame and guilt could lead to different goal-related behaviors, and since shame has been understudied in the self-regulation and self-control literature, it is worthwhile to investigate how shame, as opposed to guilt, would affect behavior after goal failure.

### 2.2. Shame Versus Guilt

Shame and guilt are both negative self-conscious emotions in the sense that they are both generalized feelings of distress that involve negative self-evaluations. However, cognitive appraisal theories of emotion predict that the same outcome can evoke very different emotional reactions depending on an individual’s beliefs about the causes of the outcome (Smith & Ellsworth, 1987; Wiener, 1980). In this regard, when a bad event occurs, the attribution that follows, be it stable or unstable, controllable or uncontrollable, can instigate different self-relevant emotions. This, in turn, leads to differences in what individuals tend to focus on after negative events occur—the self versus the behavior. Thus, shame and guilt differ in important ways, ranging from the focus of the emotion to the impact on the self to internal versus external judgments.

In guilt, the object of concern is some specific action or failure to act: “I did something bad”. Therefore, when guilty, people feel that they committed a “bad thing” (Niedenthal et al., 1994), leading to feelings of regret and remorse along with guilt over the bad thing that was performed (Tangney et al., 1992). In terms of attributions, guilt can be conceptualized as involving external, specific, controllable, and less stable attributions (Tangney et al., 2007). Guilt often arises from the understanding that one’s actions have violated external moral standards and societal rules and expectations. Therefore, when guilty, a person’s self-concept and identity remain intact, and the self remains “able” because people attribute their failure to this one-time act rather than the self. Therefore, guilt is more constructive in nature, where it acknowledges the wrongdoing while still preserving a sense of inherent worth (Duhachek et al., 2012). In this regard, it serves as a motivator for reparative action, such as making amends, confessing, apologizing, undoing, and repairing (Tangney, 1990).

With shame, on the other hand, the object of concern is the entire self. When something bad happens, it is not perceived as a bad thing; it is experienced as a reflection of a bad self, where the entire self is negatively evaluated (Tangney et al., 1992). When experiencing shame, people do not simply believe that they committed something “bad”; they feel as if they are a “bad person” (Niedenthal et al., 1994). Shame has adverse effects on one’s self-worth. Hence, shame involves internal, global, uncontrollable, and stable attributions (Tangney et al., 2007). It often arises from the perception that one has failed to live up to internal standards. The shame experience involves a considerable shift in self-perception. This corresponds to a sense of worthlessness and feeling small and powerless (Tangney, 1990). People feel powerless because they attribute their failure to the inability of the self. The motivations of ashamed people stand in sharp contrast to those of guilty people. Shame is more destructive and damaging in nature. Instead of leading to corrective action, it can lead to hiding from others, withdrawals, and feelings of inferiority. More specifically, when people feel ashamed, they want to remove themselves from the situation that has led to this experience.

When individuals fail to achieve a certain goal, the emotional experience of shame versus guilt can lead to different psychological effects. People who experience guilt are more likely to focus on specific actions or decisions that led to this failure rather than on themselves. The individual might think, “I didn’t work hard enough”, or “I made the wrong choices”, but still maintain a sense of personal worth (Meade et al., 2019). This is a constructive mindset. As such, guilt often spurs corrective action. Instead of avoiding the situation, people with feelings of guilt may review the reasons for failure, identify areas of improvement, and plan to adjust their efforts. Because guilt focuses on actions, it preserves self-worth and gives individuals a sense of control over their actions (Billore et al., 2023). In this regard, individuals who are experiencing guilt are better able to separate the act of failing from their identity. This encourages accountability and responsibility, which, in turn, supports bouncing back and trying again (Choi, 2024).

In contrast, people who experience shame as a result of goal failure are more likely to develop a deep sense of personal inadequacy, where they internalize the failure as a reflection of their inherent flaws (C. Shi et al., 2021). The motivations of ashamed individuals stand in sharp contrast to those of guilty individuals. Here, the thought may be, “I failed because I’m not good enough”, leading to lower self-esteem. This fosters a destructive loop of negative self-talk where individuals continue to see themselves as fundamentally flawed or unworthy (Stichter, 2020). Thus, people who are experiencing shame are more likely to avoid situations related to failure and disengage from future attempts to achieve their goals since any future failure could reinforce the belief that they are inherently incompetent. Shame affects one’s core identity; it can create feelings of helplessness, making it harder to bounce back or develop resilience (Stichter, 2020). In this regard, individuals experiencing shame might feel powerless to change or improve.

In sum, guilt is typically constructive, allowing the individual to reflect on their actions, learn from their experiences, and make changes for future success. It encourages a growth mindset and fosters resilience. Shame, by contrast, tends to be destructive, leading to negative self-evaluation, avoidance, withdrawal, and feelings of helplessness. It discourages future attempts and promotes a fixed, negative self-view. Despite these differences and distinct emotional responses, studies on shame in the context of goal-directed behavior remain relatively scarce, and the literature on guilt presents inconsistent findings. This study contributes to the literature by directly comparing the effects of shame and guilt, along with individual differences in emotional proneness, clarifying these inconsistencies and highlighting the role of shame in shaping behavioral outcomes.

Hence, I predict that when people feel guilty about their goal progress, they will focus on the behavior rather than themselves. Therefore, they would want to repair their wrong deeds by striving harder toward their goal, and hence, when faced with a self-control dilemma, they will choose the goal-consistent option, whereas when people feel ashamed of their goal progress, they will focus on their inability to keep on progressing toward their goal. Their self-perception is shifted, leading to an avoidance reaction, which, in turn, leads to lower goal performance, and so when faced with a self-control dilemma, they will choose the goal-inconsistent option.

**H_1A_.**  *Guilt as a result of goal failure will result in an improvement in goal performance (choose a goal-consistent option).*

**H_1B_.**  *Shame as a result of goal failure will result in deterioration of goal performance (choose a goal-inconsistent option).*

## 3. Study

### 3.1. Objective

The objective of this study is to assess whether, in goal-related behavior, feelings of shame do, in fact, lead to different actions from feelings of guilt. In this study, the interest is not in studying whether the same failure could lead to one negative emotion or the other. Rather, this study’s interest is to assess the subsequent behavior that arises from goal failure and why it differs among people. In other words, this study aims to explain why, when failing a goal, some people strive harder, and others disengage from their goals.

### 3.2. Research Design

This study employs a between-subject experimental design with two conditions (Shame vs. Guilt emotional prime). Participants were randomly assigned to one of the two emotional prime conditions. These primes were designed to elicit either shame or guilt using counterfactual thinking (Niedenthal et al., 1994). Scenario-based emotional primes were used as they provided a more consistent and controlled manipulation across participants, strengthening internal validity while reducing the effects of extraneous factors such as personal experiences and differences in memory or expression.

### 3.3. Procedure

A total of 212 undergraduates from an East Coast university participated in this study in return for course credit. First, participants were informed that this study aims to assess their behavior in common daily situations while focusing on their thought processes and their emotions. Then, they completed the Test of Self-Conscious Affect (TOSCA, Tangney et al., 1992), which presents everyday situations that people encounter in their daily lives, followed by common reactions to assess respondents’ proneness to shame or guilt. This scale was measured at the beginning of the experiment to prevent the manipulation from influencing participants’ emotional predispositions. Including this measure in this study is essential as individual differences may moderate the main effect of emotional prime on the dependent variable (Tangney, 1992). While individuals have the capacity to experience both shame and guilt, some are more prone to one than the other (Lewis, 1971). Shame proneness is generally a maladaptive affective style with failures attributed to the self, whereas guilt proneness appears to be an adaptive affective style, leading people to attribute their failure to specific actions rather than the self. Guilt primes have been shown to interact with guilt proneness such that individuals high in guilt proneness tend to indulge more when primed with guilt compared to those with low guilt proneness (Zemack-Rugar et al., 2007). However, limited research has explored the moderating effects of shame proneness in response to shame primes. This study aims to address this gap with a heightened focus on shame. Therefore, it is expected to witness a three-way interaction between emotion prime, shame proneness, and guilt proneness on the dependent variable—choice of goal-consistent (inconsistent) activity.

Then, participants were exposed to the manipulation designed to instigate either feelings of shame or guilt. Research demonstrated that counterfactual thinking has a significant effect on individuals’ emotional responses to negative events (Niedenthal et al., 1994). In a series of studies, they present participants with adverse scenarios and ask them to create counterfactual statements (statements to undo these negative scenarios). Participants who were asked to start their statements with “If only I weren’t” exhibited higher levels of shame compared to those who started their statements with “If only I hadn’t”. The latter group exhibited higher levels of guilt, indicating distinct emotional consequences resulting from self- versus behavior-focused counterfactuals. Therefore, counterfactual thinking was employed as a manipulation to prompt shame versus guilt emotional responses. Further, as previously noted, the situations that elicit shame and guilt are generally comparable (Tangney, 1992). Accordingly, all participants were presented with the same savings goal scenario adapted from Soman and Cheema (2004). Participants were requested to vividly imagine that the described scenario had actually happened to them:

*“Imagine that you have a job that pays USD 4000 monthly after taxes (i.e., take-home income). Your monthly non-discretionary expenses, like rent, bills, groceries, transportation, and insurance, add up to USD 2000. This leaves USD 2000 each month to spend on discretionary items. However, a few months ago, you decided to start saving money for the future. Taking a tip from a popular money management book, you decided to set a personal savings goal for yourself. In particular, you decided that of the USD 2000 that remains after your non-discretionary expenditures, you would like to save USD 700. In other words, you would like to spend no more than USD 1300 on discretionary items. However, you have not been so successful in achieving that goal over the past months. Now, imagine that it is almost the end of November, and you are checking your expenses, and you realize that after paying all your necessary expenses, you have only USD 500 left from your paycheck for this month, which means that again you exceeded your spending limit.”*

After reading the scenario, participants were asked to imagine ways to undo this goal failure. One group of participants was asked to start writing the paragraph with “If only I weren’t”, and the other group was asked to start writing the paragraph with “If only I haven’t”. Subjects were told the following:

*“Now complete the sentence stem “If only I were (were not) …” Complete the stem twice. Both times, describe how, if you were or were not a particular TYPE OF PERSON (e.g., with particular personality traits, emotional styles, intelligence), you wouldn’t have gotten into this situation in the first place. Thus, your sentence should take the form: “If only I were (were not) [a particular type of person].”*

The remaining participants were instructed to imagine alternative behaviors and to complete the stem as follows: *“If only I had (had not) …”* They were told to describe specific behaviors that they could have taken or not taken so that they would not have found themselves in this situation. Thus, their sentences took the form “If only I had (had not) [done something].”

Then, subjects were told that they were going to be making several different decisions that correspond to choices that many people must make in their daily lives. In this section, subjects were faced with a self-control dilemma:

*“Now, a friend calls you the next day to tell you that a local theater is selling a special package for USD 150. The package includes a music performance by an artist that you really like. The package also includes dinner and drinks. Your friend plans to go, but at the back of your mind, you are thinking about your expenses this month. You promise your friend that you will call back soon. You have a few minutes to think about this and then make a decision.”*

Then, subjects were given a choice of whether to buy the package or not, where buying the package is the goal-inconsistent behavior. This was followed by some demographic variables, including age, gender, and income. Subjects were then debriefed, thanked, and dismissed.

## 4. Results

### 4.1. Sample Characteristics

The sample consisted of 68% females and 32% males. Respondents’ ages ranged between 18 and 46, with a median age of 20. Reported annual income varied between USD 3000 and USD 800,000. Neither gender, age, nor income had a significant effect on consumer choice (all *p* > 0.1) and, thus, were dropped from further analysis.

### 4.2. Moderation Analysis

Moderation Analysis using Process Model 3 (Hayes, 2012), with emotion prime and individual emotional proneness as independent variables and goal-consistent/inconsistent choice as the dependent variable, results reveal a significant two-way interaction (*β* = −8.3744, *p* = 0.05) between prime and shame proneness indicating that proneness to shame moderates the effect of emotion prime on choice. The negative coefficient indicates that when shame proneness is high, those who are primed with shame are more likely to behave in a goal-inconsistent manner and choose to buy the indulgent choice, suggesting that shame (as a primed state or as a trait) leads to goal withdrawals and avoidance. More importantly, results also reveal a significant three-way interaction (*χ*^2^ = 3.5376, *p* = 0.06). Further analysis indicates that when shame proneness was low and guilt proneness was high, the emotion prime had a significant positive effect on choice (*β* = 0.968, *p* = 0.02, CI [0.1014, 1.83561]), leading participants to behave in a manner consistent with their goals, namely, refraining from buying the indulging package. Conversely, when shame proneness was high and guilt proneness was low (*β* = −2.0065, *p* = 0.03, CI [−3.7371, −0.2860]), the emotion prime had a negative influence on choice, such that individuals behaved in a goal-inconsistent manner and chose to buy the indulgent package. There were no other significant effects. These findings support my hypothesis that even though shame and guilt are both negatively valenced, they lead to distinct behaviors. Specifically, shame appears to be maladaptive, leading individuals to withdraw from their goals, whereas guilt is more adaptive in nature, motivating consumers to persevere in their goal pursuit and opt for corrective choices [Refer to Table 1].

**Figure 1:**
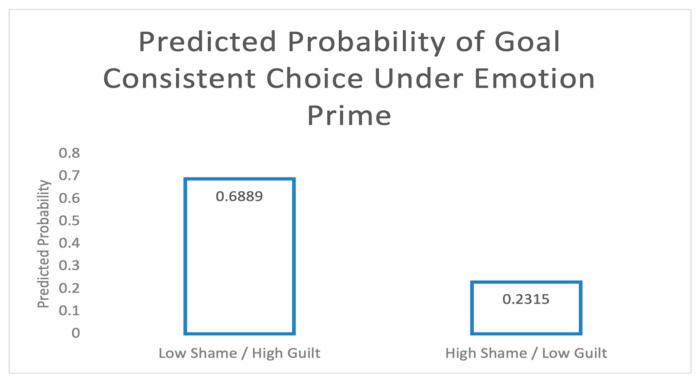
Predicted Probability of Choice Under Emotion Prime.

To illustrate these moderating effects, I plotted the predicted probabilities derived from Process Model 3 (Hayes, 2012). More specifically, I visualized the conditional effects of emotion prime (shame vs. guilt) at two key moderator points: low shame and high guilt proneness and high shame and low guilt proneness. Figure 1 shows model-estimated probabilities under these two key combinations of the moderators. The predicted probability of making a goal-consistent choice in the Low Shame/High Guilt condition was 0.69 (*p* = 0.03), whereas in the High Shame/Low Guilt condition, it was 0.23 (*p* = 0.02). These predicted probabilities highlight that guilt proneness promotes goal-consistent behavior under emotional priming, whereas shame proneness hinders it.

## 5. Discussion

This study’s findings provide empirical evidence highlighting the distinct effects of shame and guilt on goal-directed behaviors. Specifically, results indicate that these two negatively valenced self-conscious emotions lead to divergent decisions and behavioral outcomes. When individuals exhibit high guilt proneness and low shame proneness, they are more likely to engage in goal-consistent behaviors by exercising self-control and refraining from purchasing the indulgent package. Whereas individuals with high shame proneness and low guilt proneness were more likely to engage in goal-inconsistent behaviors and indulge in purchasing the package. These findings highlight the differential motivational consequences of shame and guilt, aligning with prior research that suggests that even if shame is more intense and painful than guilt (Tangney, 1992), it often leads to withdrawal and avoidance (Mróz & Sornat, 2022). In contrast, guilt fosters corrective action and persistence in goal pursuit.

This article builds on the goal-related emotions theories (Baumeister & Heatherton, 1996; Duckworth et al., 2016; Fujita, 2011; Loewenstein, 1996; Moors et al., 2021) and indulgence and self-control theories (Baumeister & Vohs, 2016; Hofmann et al., 2009; Jia, 2019) to include shame as an emotional experience that might affect choice and subsequent goal-related behavior. Even though guilt plays a central role in choices and decision-making (Shen, 2018), this study shows that shame, even though it shares the same negative valence as guilt, leads to different behaviors. Moreover, by introducing shame, this article addresses the opposing predictions of goal failures on subsequent behavior by showing that the type of the discrete emotion has a major influence on subsequent behavior rather than the valence of the general state of affect.

These findings also contribute to the literature on self-conscious emotions by reinforcing the notion that guilt serves as an adaptive emotion, fostering behaviors that align with long-term goals. Guilt-prone consumers experience a heightened motivation to repair previous transgressions by making choices that allow them to uphold their goals. Conversely, shame, previously shown to increase feelings of powerlessness and self-derogation (Stichter, 2020), undermines goal pursuit by fostering withdrawal tendencies. These results are consistent with previous literature linking shame to lower self-efficacy (C. Shi et al., 2021) and a sense of personal deficiency (Shen, 2018), which, in turn, leads them to disengage from efforts to rectify previous transgressions.

These findings also have implications for marketing strategies aimed at influencing consumer choices. I show that strategies that elicit guilt rather than shame are more effective in persuading consumers to engage in self-regulation and make goal-consistent choices. For example, rather than inducing shame toward consumers as “bad” people, public health campaigns aiming at promoting healthy lifestyles can highlight specific actions consumers can take to correct past mistakes. In a similar vein, companies promoting prosocial behaviors, such as sustainable consumption, may benefit from guilt-induced messages focusing on reparative actions as opposed to shame appeals focusing on the negative evaluation of the self (e.g., you are a bad person for not purchasing sustainable products).

Despite these contributions, some concerns still remain. First, this study uses a hypothetical task. Therefore, the results capture what participants believe that they would do rather than what they will actually do. Future research could extend these findings through field-based studies that test how shame- and guilt-based strategies influence actual consumer behavior in real-world marketing or social media settings. Second, our study relied on self-reported measures of guilt and shame proneness, which may not fully capture the complexity of these emotions. Further, it is important to extend the generalizability of the findings by including different scenarios with different self-control dilemmas, such as academic performance, interpersonal relationships, and sustainable and ethical lifestyles. Future studies should explore this phenomenon in these different contexts of behaviors, not only as opposed to each other but as opposed to neutral or positive emotions.

Furthermore, future research should investigate the mediating effect of self-efficacy, an individual’s perceived level of ability (Elliott & Dweck, 1988). Specifically, individuals who are more prone to experience shame in response to their failure to progress toward a certain goal are more likely to perceive lower levels of self-efficacy compared to those who experience guilt. This diminished sense of ability, in turn, leads to further deterioration in goal performance.

Finally, regulatory-focus theory (Higgins, 2002) distinguishes between two motivational orientations—promotion-focused and prevention-focused. For instance, an individual pursuing a goal to be more sustainable who purchases sustainable products is likely to be promotion-oriented, approaching positive outcomes and goal attainment. On the other hand, someone who focuses on avoiding unsustainable products is likely to be prevention-oriented, aiming to minimize negative outcomes. Future research could explore how shame would interact with these two regulatory focus orientations. In this context, goal failure could stem from failing to engage in positive actions (e.g., not buying sustainable products) or from engaging in harmful behaviors (e.g., littering).

In conclusion, this research sheds light on an important yet unexplored aspect of goal-related emotions and behaviors. My findings underscore the critical distinction between shame and guilt in shaping consumer decision-making processes in the context of goal pursuit. Results indicate that, in addition to guilt, shame could also be the result of a certain goal failure, and it has a different influence on subsequent goal-related behavior. In this way, the present article shows that more negatively intense and painful feelings do not always lead to goal improvement and more goal-consistent behavior. On the contrary, they could also lead to deterioration in performance and goal-inconsistent choices. In this regard, shame fosters maladaptive withdrawal tendencies that may undermine self-control. These insights not only contribute to the literature on self-conscious emotions and goal attainment but also offer managers a guide for designing marketing campaigns that harness the motivational power of guilt to promote positive behavioral changes.

## Figures and Tables

**Table 1:** Descriptive Statistics and Conditional Effects from Moderation Analysis.

Variable/Condition	Results	Interpretation
Gender Distribution	68% female, 32% male	Sample skewed female, no significant effect on choice (*p* = 0.2)
Age Range (Median)	18–46 years (Median = 20)	Young sample, no significant effect on choice (*p* = 0.4)
Income Range	$3000–$800,000	Wide income range, no significant effect on choice (*p* = 0.7)
Prime × Shame Proneness	*β* = −8.3744, *p* = 0.05	Shame proneness significantly moderates the effect of emotion prime on choice
Prime × Shame Proneness × Guilt Proneness	*χ*^2^ = 3.5376, *p* = 0.06	Significant three-way interaction—suggests a joint role of shame and guilt in choice
Low Shame/High Guilt	*Predicted Probability* = 0.69, *p* = 0.03	High guilt and low shame promote goal-consistent behavior
High Shame/Low Guilt	*Predicted Probability* = 0.23, *p* = 0.02	High shame and low guilt promote goal-inconsistent behavior

## Data Availability

The datasets generated during and/or analyzed during the current study are available from the corresponding author upon reasonable request.
